# Improved prediction of fracture risk leveraging a genome-wide polygenic risk score

**DOI:** 10.1186/s13073-021-00838-6

**Published:** 2021-02-03

**Authors:** Tianyuan Lu, Vincenzo Forgetta, Julyan Keller-Baruch, Maria Nethander, Derrick Bennett, Marie Forest, Sahir Bhatnagar, Robin G. Walters, Kuang Lin, Zhengming Chen, Liming Li, Magnus Karlsson, Dan Mellström, Eric Orwoll, Eugene V. McCloskey, John A. Kanis, William D. Leslie, Robert J. Clarke, Claes Ohlsson, Celia M. T. Greenwood, J. Brent Richards

**Affiliations:** 1 Centre for Clinical Epidemiology, Lady Davis Institute for Medical Research, Jewish General Hospital, Room H-413, 3755 Chemin de la Côte-Sainte-Catherine, Montreal, Quebec H3T 1E2 Canada; 2 Quantitative Life Sciences Program, McGill University, Montreal, Canada; 3 Centre for Bone and Arthritis Research, Department of Internal Medicine and Clinical Nutrition, Institute of Medicine, Sahlgrenska Academy, University of Gothenburg, Gothenburg, Sweden; 4 Bioinformatics Core Facility, Sahlgrenska Academy, University of Gothenburg, Gothenburg, Sweden; 5 Clinical Trial Service Unit and Epidemiological Studies Unit, Nuffield Department of Population Health, University of Oxford, Oxford, UK; 6 National Institute for Health Research Oxford Biomedical Research Centre, Oxford University Hospitals NHS Foundation Trust, Oxford, UK; 7 Department of Epidemiology, Biostatistics and Occupational Health, McGill University, Montreal, Canada; 8 Department of Diagnostic Radiology, McGill University, Montreal, Canada; 9 MRC Population Health Research Unit, University of Oxford, Oxford, UK; 10 School of Public Health, Peking University Health Science Centre, Beijing, China; 11 Clinical and Molecular Osteoporosis Research Unit, Department of Orthopedics and Clinical Sciences, Lund University, Lund, Sweden; 12 Skåne University Hospital, Malmö, Sweden; 13 Bone & Mineral Unit, Oregon Health & Science University, Portland, USA; 14 Department of Medicine, Oregon Health & Science University, Portland, USA; 15 Mellanby Centre for Bone Research, Centre for Integrated Research in Musculoskeletal Ageing, University of Sheffield, Sheffield, UK; 16 Centre for Metabolic Bone Diseases, University of Sheffield, Sheffield, UK; 17 Mary Mackillop Institute for Health Research, Australian Catholic University, Melbourne, Australia; 18 Department of Medicine, University of Manitoba, Winnipeg, Canada; 19 Department of Drug Treatment, Sahlgrenska University Hospital, Gothenburg, Sweden; 20 Department of Human Genetics, McGill University, Montreal, Canada; 21 Gerald Bronfman Department of Oncology, McGill University, Montreal, Canada; 22 Department of Twin Research and Genetic Epidemiology, King’s College London, London, UK

## Abstract

**Background:**

Accurately quantifying the risk of osteoporotic fracture is important for directing appropriate clinical interventions. While skeletal measures such as heel quantitative speed of sound (SOS) and dual-energy X-ray absorptiometry bone mineral density are able to predict the risk of osteoporotic fracture, the utility of such measurements is subject to the availability of equipment and human resources. Using data from 341,449 individuals of white British ancestry, we previously developed a genome-wide polygenic risk score (PRS), called gSOS, that captured 25.0% of the total variance in SOS. Here, we test whether gSOS can improve fracture risk prediction.

**Methods:**

We examined the predictive power of gSOS in five genome-wide genotyped cohorts, including 90,172 individuals of European ancestry and 25,034 individuals of Asian ancestry. We calculated gSOS for each individual and tested for the association between gSOS and incident major osteoporotic fracture and hip fracture. We tested whether adding gSOS to the risk prediction models had added value over models using other commonly used clinical risk factors.

**Results:**

A standard deviation decrease in gSOS was associated with an increased odds of incident major osteoporotic fracture in populations of European ancestry, with odds ratios ranging from 1.35 to 1.46 in four cohorts. It was also associated with a 1.26-fold (95% confidence interval (CI) 1.13–1.41) increased odds of incident major osteoporotic fracture in the Asian population. We demonstrated that gSOS was more predictive of incident major osteoporotic fracture (area under the receiver operating characteristic curve (AUROC) = 0.734; 95% CI 0.727–0.740) and incident hip fracture (AUROC = 0.798; 95% CI 0.791–0.805) than most traditional clinical risk factors, including prior fracture, use of corticosteroids, rheumatoid arthritis, and smoking. We also showed that adding gSOS to the Fracture Risk Assessment Tool (FRAX) could refine the risk prediction with a positive net reclassification index ranging from 0.024 to 0.072.

**Conclusions:**

We generated and validated a PRS for SOS which was associated with the risk of fracture. This score was more strongly associated with the risk of fracture than many clinical risk factors and provided an improvement in risk prediction. gSOS should be explored as a tool to improve risk stratification to identify individuals at high risk of fracture.

**Supplementary Information:**

The online version contains supplementary material available at 10.1186/s13073-021-00838-6.

## Background

Osteoporosis is among the most common diseases worldwide and is characterized by a reduction in bone mass and microarchitectural integrity, resulting in an increased predisposition to fracture [1]. In the Western world, between one third to one half of women will experience an osteoporotic fracture in their lifetime [2]. The burden of this disease will increase substantially as our population ages, such that by the year 2040, the annual incidence of hip fractures is expected to surpass 500,000 in the USA and the direct medical costs of surgery for each hip fracture will exceed $65–68,000 USD [3]. Given this impending financial and societal burden, efforts to improve earlier detection of those at high risk of fracture will enable more effective intervention strategies.

Studies based on large cohorts have shown that skeletal measures, such as quantitative ultrasound speed of sound (SOS) and dual-energy X-ray absorptiometry (DXA) bone mineral density (BMD), can predict the risk of osteoporotic fracture with a high specificity but poor sensitivity [1, 4]. However, the utility of such measurements is subject to the availability of equipment and human resources and, in some jurisdictions, can be relatively expensive [5]. Effective risk stratification relying on other clinically relevant risk factors is available. For example, the Fracture Risk Assessment Tool (FRAX) has been developed to predict 10-year probabilities of major osteoporotic fracture and hip fracture by integrating multiple clinical risk factors [6]. Combining these clinical risk factors with femoral neck BMD in the FRAX score has become widely accepted as a method to appropriately stratify individuals for therapy [7–10].

With the advent of large-scale genome-wide genotyped cohort resources, such as UK Biobank, and emerging computational algorithms, genomic prediction of complex traits and diseases may improve disease prediction accuracy and thus potential clinical utility [11, 12]. Both BMD and SOS are clinically used to predict fracture risk and are moderately correlated, with high heritability (50–85%) [13]. This suggests that polygenic risk scores could be developed for these skeletal measures [14, 15].

Polygenic risk scores could also be developed in the field of osteoporosis to better identify individuals at risk for fracture. In previous work, a genetic risk score including 21 single nucleotide polymorphisms (SNPs) in 19 genes associated with osteoporosis-related traits was moderately associated with nonvertebral fracture risk in Korean postmenopausal women and might slightly improve clinical osteoporotic risk factor-based fracture risk prediction [16]. Another genetic risk score based on 62 SNPs associated with femur neck or lumbar spine BMD accounted for approximately 2% of the variance in BMD and conferred a hazard ratio of 1.20 for incident fragility fracture per one standard deviation increase [17]. A similar score summarizing 63 BMD loci was associated with BMD during childhood and was moderately associated with fracture risk in adulthood [18, 19]. Moreover, a genetic risk score leveraging 979 SNPs in 74 osteoporosis-susceptibility genes was developed in Japanese women and could identify individuals having prevalent vertebral fractures with an area under the receiver operating characteristic curve (AUROC) of 0.788 [20]. More recently, using 1103 independent SNPs associated with estimated BMD (eBMD) of the heel, a polygenic risk score for eBMD has been shown to be able to predict site-specific fractures [21]. However, due to the polygenic nature of these skeletal traits and the usually limited study sample sizes, existing genetic risk scores may not be able to sufficiently capture the underlying genetic predisposition towards fracture and therefore may not provide optimal predictive performance. Consequently, the clinical utility of genetic risk scores in fracture risk prediction, especially among the elderly population, has been questioned [22].

Using data from the UK Biobank, we have previously developed a genome-wide polygenic risk score for SOS, called genomic SOS (gSOS), that captures 25.0% of the total variance in SOS [23]. This largely exceeded an existing polygenic risk score which explained 17.2% of the total variance and achieved an AUROC of 0.570 for identifying individuals with any type of fracture [24]. In comparison, FRAX clinical risk factors, age, sex, and BMI together explain 4.0% (95% confidence interval (CI) 3.7–4.2%) of the variance in SOS [23]. gSOS has been shown to be independent of other commonly used clinical risk factors and significantly associated with fracture risk [23]. However, the clinical utility of gSOS to predict osteoporotic fracture risk remains largely unexplored. Here, we have assessed the predictive performance of gSOS in 90,172 individuals of European ancestry in four study cohorts, the UK Biobank [25], the United States-based Osteoporotic Fractures in Men Study (MrOS US) [26], the Sweden-based Osteoporotic Fractures in Men Study (MrOS Sweden) [27], and the Study of Osteoporotic Fractures (SOF) [28, 29]. We further investigated whether combining gSOS with FRAX could better identify individuals at high risk of osteoporotic fracture. We also tested the generalizability of gSOS to a non-European population consisting of 25,034 individuals, using data from the China Kadoorie Biobank (CKB) study [30]. Data from this study have been presented at the American Society for Bone and Mineral Research 2020 Annual Meeting [31].

## Methods

### Study cohorts

This study included five cohorts: the UK Biobank [25], the MrOS US [26], the MrOS Sweden [27], the SOF [28], and the CKB [30].

The UK Biobank comprises more than 500,000 participants who were recruited at multiple assessment centers in the UK and were enrolled between 2006 and 2010 at ages ranging from 40 to 69 years. Compared to a general population, the UK Biobank has been reported to be healthier, less obese, and less likely to smoke and drink alcohol [32]. Participants of the UK Biobank were genome-wide genotyped using Affymetrix arrays, and their genotypes were imputed to the Haplotype Reference Consortium (HRC) reference panel [25, 33]. Site-specific major osteoporotic fractures were defined based on ICD10 codes of primary diagnoses (M8002, M8003, M8005, M8008, M8082, M8083, M8085, M8088, S220, S320, S422, S423, S424, S520, S521, S522, S523, S525, S526, S529, S720, S721, and S722) and self-reported medical history. Since the exact cause of fracture was not identifiable, these identified events may include traumatic fractures. Fracture risk factors were those captured at the baseline visit, and incident fractures were defined to be those occurring after the baseline visit.

The MrOS is an international multicenter longitudinal study comprised of elderly men [27]. The MrOS US cohort recruited 5995 men aged ≥ 65 years at multiple assessment centers in the USA between 2000 and 2002 [26]. Baseline examinations were conducted upon recruitment followed by a more extensive questionnaire after 2–2.5 years of follow-up. After a mean follow-up of 4.5 years, a second clinic visit was completed. Tri-annual questionnaires inquiring about the occurrence of incident falls, fractures, and fracture risk factors were collected during the intervening period. In this cohort, 11% of the participants were visible minorities. Among these 5995 men, 5130 were genotyped. After overlaying the first two genetic principal components with those from the 1000 Genomes Project [34], 4663 men of a genetically determined European ancestry were included in this study. Their genotypes were imputed to the HRC reference panel. The MrOS Sweden cohort recruited 3014 men aged 69 to 81 years in three Swedish cities between 2001 and 2004 [35]. After baseline examinations, participants were followed for up to 10 years after the baseline examination. Fracture evaluation was done by searching digital X-ray archives and matching them with MrOS Sweden participants using unique personal identification number, which all Swedish citizens have. Among these 3014 men, 1880 were genotyped and included in this study. Their genotypes were imputed to the HRC reference panel.

The SOF cohort recruited 9704 women at four assessment centers in the USA who were aged ≥ 65 years at enrollment between 1986 and 1988 [28]. Falls and fractures were monitored every 4 months. After baseline examinations, follow-up examinations took place approximately every 2 years during a mean follow-up of 14.5 years. Among these 9704 women, 3625 were genotyped, and 3615 women with a genetically determined European ancestry with reference to the 1000 Genomes Project [34] were included in this study. Their genotypes were also imputed to the HRC reference panel.

To be eligible for the MrOS US, the MrOS Sweden, or the SOF cohorts, participants were required to (1) be able to walk without the assistance of another, (2) not have had bilateral hip replacements, and (3) be able to provide self-reported data [26–28]. All reported fractures after baseline were confirmed by a physician review of the radiology report.

The CKB cohort recruited more than 500,000 adults aged between 30 and 79 years, from 2004 to 2008 in 10 survey sites in China [30]. After the baseline survey, long-term follow-up was conducted by accessing electronic records of mortality registries, morbidity registries, and all hospitalized events and procedures that are available for 98% of the participants covered by the nation-wide health insurance system. SOS and incident fracture were collected for 25,034 participants during the second survey of the CKB between 2013 and 2014. These participants were genotyped, and their genotypes were imputed to a 1000 Genomes Project-based reference panel for East Asian populations [34].

Each study was approved by the institutional review boards of participating institutions and, all participants provided informed consent to respective studies.

### Risk factor measurement

Clinically relevant risk factors were measured at the baseline visit for these cohorts, including FRAX clinical risk factors, such as age, sex, BMI (in the unit of kg/m^2^), prior fractures (hip fractures and other osteoporotic fractures), smoking, glucocorticoid use, rheumatoid arthritis, and femoral neck BMD based on DXA scan. Diagnosis of secondary causes of osteoporosis (type 1 diabetes, osteogenesis imperfecta in adults, untreated long-standing hyperthyroidism, hypogonadism or premature menopause, chronic malnutrition, or malabsorption and chronic liver disease) was available only in the UK Biobank. Parental history of fracture, at-risk drinking (≥ 3 units per day), and self-reported falls (a risk factor independent of FRAX probability [36]) based on interviews or questionnaires were available in the MrOS US, the MrOS Sweden, and the SOF cohorts. Cohorts with missing risk factor measurements were considered free of the corresponding risk factors for the derivation of FRAX probability, as suggested by the FRAX model (https://www.sheffield.ac.uk/FRAX/faq.aspx).

### Development of a polygenic risk score

As described previously [23], we developed a polygenic risk score using a statistical learning approach to predict SOS, a risk factor for osteoporotic fracture in the UK Biobank. The polygenic risk score for FRAX is referred to here as “gSOS.”

There were 426,811 participants in the UK Biobank of white British ancestry who had measured SOS and had undergone genome-wide genotyping. We first assigned these individuals to a training dataset (*N* = 341,449, 80% of the total cohort), a model selection dataset (*N* = 5335, 1.25% of the total cohort), and a test dataset (*N* = 80,014, 18.75% of the total cohort). All 4717 individuals with femoral neck BMD measurements were assigned to the test dataset in order to compare the fracture predictive performance of gSOS with BMD. We performed a genome-wide association study on the training dataset using a linear mixed model adjusting for age, sex, assessment center, genotyping array, and the top 20 genetic principal components. We next performed a series of least absolute shrinkage and selection operator (LASSO) regressions [37] in the training dataset using SOS as an outcome and SNPs as predictors, applying different *p* value thresholds (*p* ≤ 5 × 10^−3^, *p* ≤ 5 × 10^−4^, *p* ≤ 5 × 10^−5^, *p* ≤ 5 × 10^−6^, *p* ≤ 5 × 10^−7^, and *p* ≤ 5 × 10^−8^) after removing SNPs demonstrating linkage disequilibrium (*r*^2^ > 0.05) with other SNPs. Each LASSO regression model thus yielded a polygenic risk score with tuned coefficients associated with different subsets of SNPs. We selected the best-performing model as that with the highest proportion of variance explained in the model selection dataset (Additional file 1: Table S1). This model included 21,717 activated SNPs with a *p* value ≤ 5 × 10^−4^ and was able to explain 23.18% (95% CI 22.66%–23.69%) of the total variance in the test dataset [23]. Since all SNPs employed in the optimized polygenic risk score were present in all European ancestry cohorts under investigation, we obtained a genetically predicted SOS (gSOS) for each individual in the UK Biobank test dataset as well as the other three cohorts. We did not perform downstream analyses on the UK Biobank training and model selection datasets, since these datasets were used to generate and select gSOS, respectively, and could therefore be prone to biased estimates due to model over-fitting.

Because the genotypes of CKB participants were imputed to a different reference panel, only 13,848 activated SNPs were available in the CKB. Using these available SNPs, we derived a gSOS estimate for each CKB participant. Differences in minor allele frequencies between the CKB and the UK Biobank are summarized in Additional file 1: Fig. S1.

### Derivation of FRAX-based risk scores

The country-specific FRAX tool (https://www.sheffield.ac.uk/FRAX/) incorporating clinically relevant risk factors was used to calculate the 10-year probability of experiencing a major osteoporotic fracture or a hip fracture [6]. For individuals whose femoral neck BMD was available, a FRAX score including BMD was also calculated using this algorithm.

We also developed a clinical risk factor plus gSOS-based FRAX (FRAX-gSOS). The largest meta-analysis of osteoporosis cohorts to date, which did not include the UK Biobank, estimated that one standard deviation decrease in SOS was associated with 1.42-fold increased odds of experiencing major osteoporotic fracture [4]. Therefore, to generate a gSOS-adjusted FRAX score for major osteoporotic fracture, we first converted the FRAX algorithm-derived fracture probabilities to odds of fracture. We divided the odds by 1.42 to the power of standardized gSOS, such that an individual with a standardized gSOS of 1 would have a 1.42-fold decreased odds; an individual with a standardized gSOS of − 1 would have a 1.42-fold increased odds. The adjusted odds were then converted back to fracture probabilities. For hip fractures, the same transformation was performed, except that the increase in risk per standard deviation decrease in SOS was previously demonstrated to be 1.80-fold [4].

Thus, for each individual, three FRAX scores were generated: a clinical risk factor-based FRAX (FRAX-CRF), a clinical risk factor plus BMD-based FRAX (FRAX-BMD), and a FRAX-gSOS. This allowed a direct comparison of the predictive accuracy of each of these three methods.

Due to the missingness of several FRAX clinical risk factors in the CKB cohort (Table 1), we did not generate FRAX scores for CKB participants. Analyses were thus restricted to testing the association (described below) between gSOS and incident major osteoporotic fracture or hip fracture risk adjusted for age and sex in the CKB cohort. We then compared the effect size of gSOS with those obtained in European populations based on the other cohorts.

**Table 1:** Study characteristics

Characteristic	UK Biobank test dataset		MrOS US		MrOS Sweden		SOF		CKB	
	Mean/*N*	Missing/*N*	Mean/*N*	Missing/*N*	Mean/*N*	Missing/*N*	Mean/*N*	Missing/*N*	Mean/*N*	Missing/*N*
Sample size	80,014		4663		1880		3615		25,034	
Baseline age (SD)	56.8 (8.0)		74.0 (6.0)		75.4 (3.2)		71.5 (5.3)		53.7 (11.0)	
Women (%)	43,723 (54.6)		0		0		3615 (100)		14,334 (57.3)	
Baseline BMI (SD)	27.3 (4.7)	4	27.4 (3.8)	1	26.4 (3.5)		26.7 (4.6)		23.7 (3.5)	
Prior fracture (%)	8008 (10.0)	21	1087 (23.3)	4	637 (33.9)	1	1274 (35.4)	17	2290 (9.2)	
Smoking (%)	6908 (8.6)		146 (3.1)	1	178 (9.5)	6	285 (7.9)	13	7957 (31.8)	
Corticosteroids use (%)	923 (1.2)		98 (2.2)	164	34 (1.8)	6	387 (10.9)	62		25,034
Rheumatoid arthritis (%)	758 (1.0)		226 (4.8)		27 (1.5)	23	252 (7.1)	74	572 (2.3)	
Parental fracture (%)		80,014	600 (16.8)	1098	164 (13.7)	686	424 (14.3)	646		25,034
At-risk drinking* (%)		80,014	182 (3.9)	6	52 (2.8)	27	102 (2.8)	1		25,034
Baseline falls^§^ (%)		80,014	984 (21.1)		298 (15.9)	6	1021 (28.3)	6		25,034
Secondary osteoporosis (%)	3352 (4.2)			4663		1880		3615		25,034
FRAX for MOF (SD)	5.1 (3.1)		9.5 (4.7)	1	13.1 (5.6)		18.7 (9.5)			
FRAX for hip fracture (SD)	0.7 (0.9)		3.8 (3.8)	1	7.0 (4.9)		6.4 (7.0)			
FRAX-gSOS for MOF (SD)	5.4 (3.9)		10.1 (6.0)	6	13.7 (7.3)		19.3 (11.2)	38		
FRAX-gSOS for hip fracture (SD)	0.9 (1.2)		4.2 (4.8)	6	7.7 (6.5)		7.0 (8.1)	38		
FRAX-BMD for MOF (SD)	4.9 (2.6)	75,273	8.1 (4.4)	2	11.1 (6.3)	19	17.1 (9.5)	151		
FRAX-BMD for hip fracture (SD)	0.7 (1.0)	75,273	2.5 (3.3)	2	5.3 (5.5)	19	5.0 (6.7)	151		
Incident major osteoporotic fracture (incidence proportion)	1189 (1.5)		560 (12.0)		337 (17.9)		707 (20.6)		323 (1.3)	
Incident hip fracture (incidence proportion)	209 (0.3)		273 (5.9)		129 (6.9)		556 (15.6)		67 (0.3)	

*Though alcohol use is available in the UK Biobank and the CKB, no information regarding the frequency of drinking to determine at-risk drinking (≥ 3 drinks per day)

^§^The UK Biobank only records self-reported falls in the past 12 months at the baseline visit

### Statistical analysis in European ancestry-based cohorts

We first standardized gSOS scores for each cohort separately to have a mean of zero and a standard deviation of one. This enables a more standardized comparison of effects across cohorts. Since risk predictors are more useful if they are independent of known risk factors, we assessed linear correlations between gSOS and clinically relevant risk factors of fracture using the Pearson correlation coefficient. We next quantified incident major osteoporotic fractures and binomial proportion CIs among individuals grouped with respect to different quantile ranges of gSOS: ≤ 1%, 1–5%, 5–20%, 20–40%, 40–60%, 60–80%, 80–95%, 95–99%, and > 99%.

We tested the association between the risk of incident major osteoporotic fracture and gSOS as well as other clinically relevant risk factors using logistic regression adjusted for age and sex. We also tested the association between the risk of incident major osteoporotic fracture and each of the three FRAX scores using logistic regression without adjusting for age and sex since they are included in the FRAX algorithm. Model comparison was performed by the likelihood ratio test. We assessed the predictive performance of each of the three FRAX scores using the AUROC or the area under the precision-recall curve (AUPRC) for major osteoporotic fractures. AUROC and CIs were computed using the R package “pROC” version 1.15.3 [38]. DeLong’s test was performed to compare AUROC [39]. AUPRC and bootstrapped CIs were computed using the R package “PRROC” version 1.3.1 [40].

Further, we evaluated the cumulative incidence of major osteoporotic fracture in the MrOS US and SOF cohorts separately by Kaplan-Meier estimates, censored at 90 years. These studies were selected because of their longer length of follow-up. We also assessed the cumulative risk of incident major osteoporotic fracture associated with gSOS, FRAX clinical risk factors, and FRAX-based scores by Cox proportional hazards regression using age at the time of fracture as the time scale. Timing of fracture was not available for other cohorts. The MrOS US and SOF cohorts were combined in the Cox models which were then sex-stratified. We assessed the predictive performance of each model using C-index (a generalization of the AUROC considering censored data in Cox models). Survival analyses were performed using the R package “rms” version 5.1-3.1 [41, 42]. C-indices and 95% CIs were computed using the R package “Hmisc” version 4.2-0 [41, 43]. The above analyses were repeated for incident hip fracture.

Lastly, we examined whether a FRAX-gSOS score can improve clinical screening by net reclassification index (NRI) and integrated discrimination index (IDI) [44]. The NRI measures how well a new predictive model correctly categorizes individuals into their corresponding groups, while the IDI quantifies changes in the average discriminative power. We compared the gSOS-FRAX score to the CRF-FRAX score. The clinical cutoff was set at 20% and 3% (above which pharmacological treatment is recommended by the National Osteoporosis Foundation [45]) respectively for predicted 10-year major osteoporotic fracture risk and hip fracture risk. NRIs and IDIs were computed using the R package “PredictABEL” version 1.2-2 [46].

## Results

### Study characteristics

Demographic features, incidence of major osteoporotic fracture and hip fracture, prevalence of each FRAX risk factor, and FRAX-based scores are summarized for each of the five cohorts used in Table 1. Among the European ancestry cohorts, the MrOS US, the MrOS Sweden, and the SOF cohorts comprised elderly men or women, while individuals in the UK Biobank test dataset were comparatively younger with lower FRAX scores. Like the UK Biobank, the CKB cohort was also younger and prior fracture, an age-related risk factor, was less prevalent in these two cohorts. In contrast, the SOF cohort, comprised entirely of elderly women, had the highest prevalence of prior fracture, corticosteroids use, and rheumatoid arthritis, as well as the highest average FRAX-based scores. Thus, the five cohorts analyzed provide different populations within which to evaluate fracture prediction performance, providing a more thorough assessment of its generalizability.

### gSOS is largely independent of clinical risk factors

gSOS demonstrated negligible correlations with FRAX clinical risk factors examined, and this lack of correlation was observed in each cohort separately (Additional file 1: Table S2). However, there was a correlation between gSOS and prior fracture (Pearson correlation ranged from − 0.12 to − 0.07 across different European ancestry cohorts and was − 0.05 in the CKB cohort). These findings suggest that gSOS might be a predictor of incident fracture, independent of FRAX clinical risk factors.

### Extremes of gSOS have large differences in incidence of major osteoporotic fracture and hip fracture

**Fig. 1:**
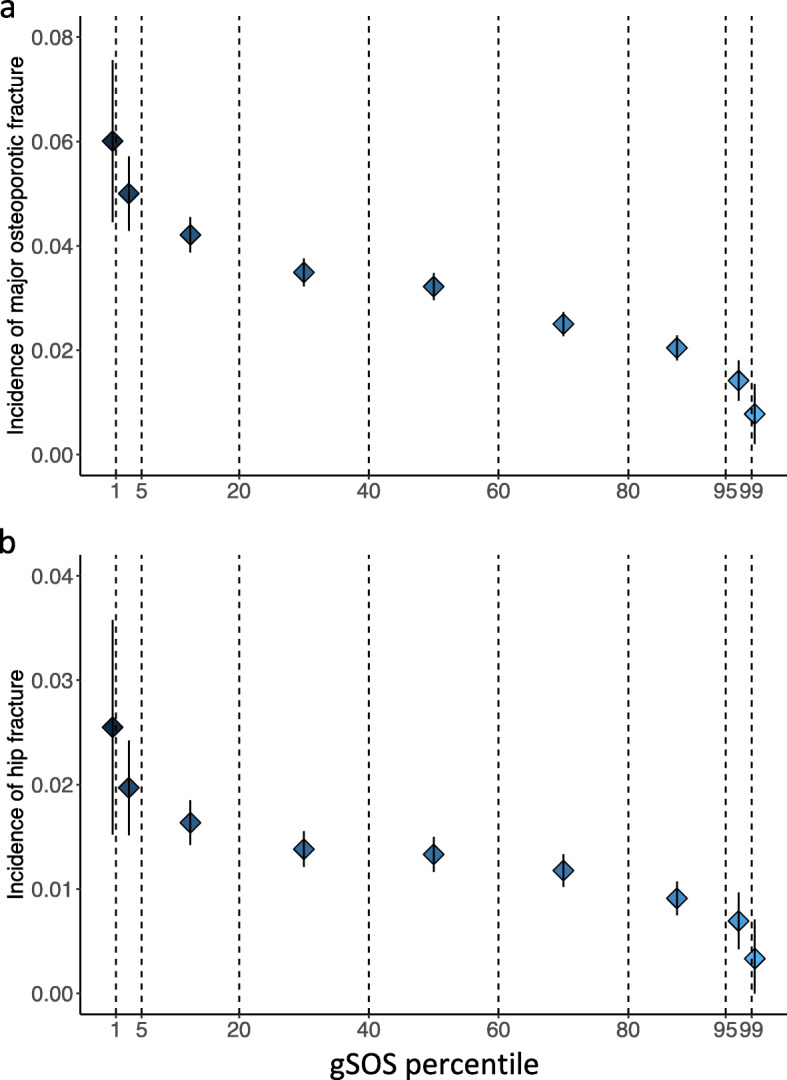
Higher incidence of **a** major osteoporotic fracture and **b** hip fracture is associated with lower gSOS. All 90,172 individuals from four European ancestry cohorts were grouped into nine groups with respect to gSOS percentiles: ≤ 1%, 1–5%, 5–20%, 20–40%, 40–60%, 60–80%, 80–95%, 95–99%, and > 99%. Diamonds denote observed incidence in each gSOS group with darker colors representing higher incidence. Vertical lines denote corresponding binomial proportion CIs. Summaries of cohort-specific incidence in each gSOS group are provided in Additional file 1: Table S2

In all European ancestry cohorts, a lower gSOS, which predicts a lower SOS, was associated with a higher incidence of major osteoporotic and hip fractures (Fig. 1a, b). For example, among individuals with a gSOS below the first percentile (1%), 6.0% (95% CI 4.5–7.6%) experienced an incident major osteoporotic fracture and 2.5% (95% CI 1.5–3.6%) experienced an incident hip fracture. In contrast, the incidence was only 0.7% (95% CI 0.2–1.4%) and 0.3% (95% CI 0–0.7%) for major osteoporotic fracture and hip fracture, respectively, among individuals with a gSOS above the 99th percentile. This represents an 8.6-fold and 8.3-fold higher incidence of major osteoporotic and hip fracture, respectively. Similar or more pronounced risk stratifications were achieved in all cohorts; the discrepancies were even larger among older individuals in the MrOS US, the MrOS Sweden, and the SOF cohorts (Additional file 1: Table S3). Overall, individuals who experienced incident major osteoporotic fracture or hip fracture had lower gSOS (Additional file 1: Fig. S2).

### gSOS was more strongly associated with fracture risk than most clinical risk factors in European populations

To compare the strength of gSOS as a predictor of fracture to standard risk factors used clinically and in the FRAX score, we chose a series of cut-points to binarize gSOS while matching the frequency of each clinical risk factor. For example, 24.2% of the population across the four European ancestry cohorts reported falls. We therefore identified individuals in the lowest 25.0% of the distribution of gSOS. We then examined whether the association between fracture and falls was stronger than that between fracture and having a gSOS among the 25% lowest in the population.

**Fig. 2:**
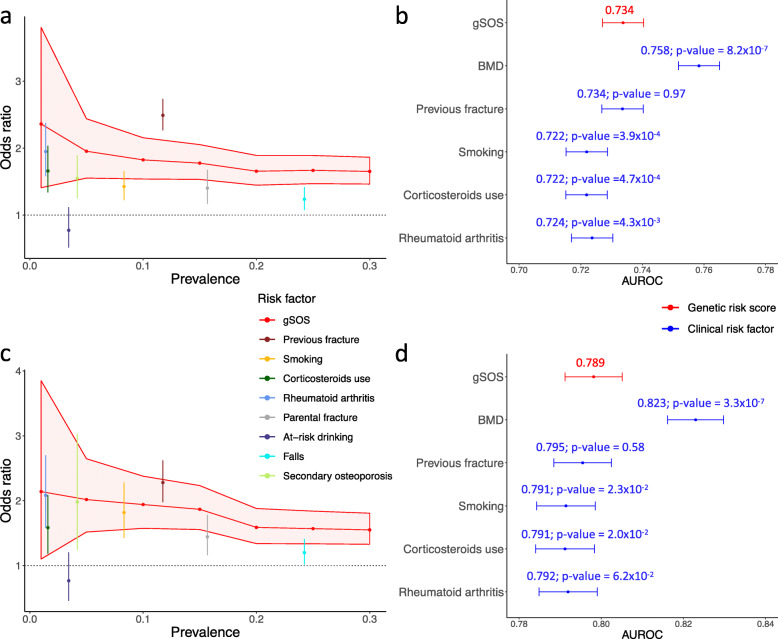
gSOS is more strongly associated with fracture risk than most clinical risk factors. **a** Comparison of associations between incident major osteoporotic fracture risk and risk factors. Different thresholds of gSOS were selected such that a similar proportion of the population would be affected by one of the clinical risk factors. **b** Comparison of the predictive performance of gSOS (red) and clinical risk factors (blue) in identifying individuals that experienced incident major osteoporotic fracture. **c** Comparison of associations between incident hip fracture risk and risk factors. **d** Comparison of the predictive performance of gSOS (red) and clinical risk factors (blue) in identifying individuals that experienced incident hip fracture. Comparison of each clinical risk factor AUROC to gSOS AUROC was based on DeLong’s test; *p* values are presented in **b** and **d**. All association tests were based on logistic regression models, adjusted for age and sex in individuals from four European ancestry cohorts. Only clinical risk factors available in all cohorts are presented in **b** and **d**, based on 12,359 individuals with complete data. 95% CIs of AUROC were derived from 200 bootstrap replicates. Summaries of the cohort-specific predictive performance of all risk factors are provided in Additional file 1: Table S4

After aligning prevalence rates of risk factors, binarized gSOS indicators had a stronger association with the odds of major osteoporotic fracture and hip fracture, than most of the other clinical risk factors, except for prior fracture (Fig. 2a, c). For instance, 25.0% of the population with the lowest gSOS had a 1.68-fold (95% CI 1.47–1.89) increased odds of major osteoporotic fracture and a 1.57-fold (95% CI 1.34–1.84) increased odds of hip fracture, whereas falls affected 24.2% of the population, but only increased the major osteoporotic fracture risk by 1.24-fold (95% CI 1.08–1.42) and the hip fracture risk by 1.20-fold (95% CI 1.02–1.41). We note, however, that the 95% CIs of these risk factors overlapped to some degree. In addition, a continuous gSOS exhibited moderately enhanced or similar predictive power for incident fracture, as measured by the AUROC (0.734 (95% CI 0.727–0.740) for incident major osteoporotic fracture; 0.798 (95% CI 0.791–0.805) for incident hip fracture) as other clinically relevant risk factors (Fig. 2b, d; Additional file 1: Table S4), except measured femoral neck BMD. This enhanced performance was consistent across all study cohorts (Additional file 1: Table S4).

**Fig. 3:**
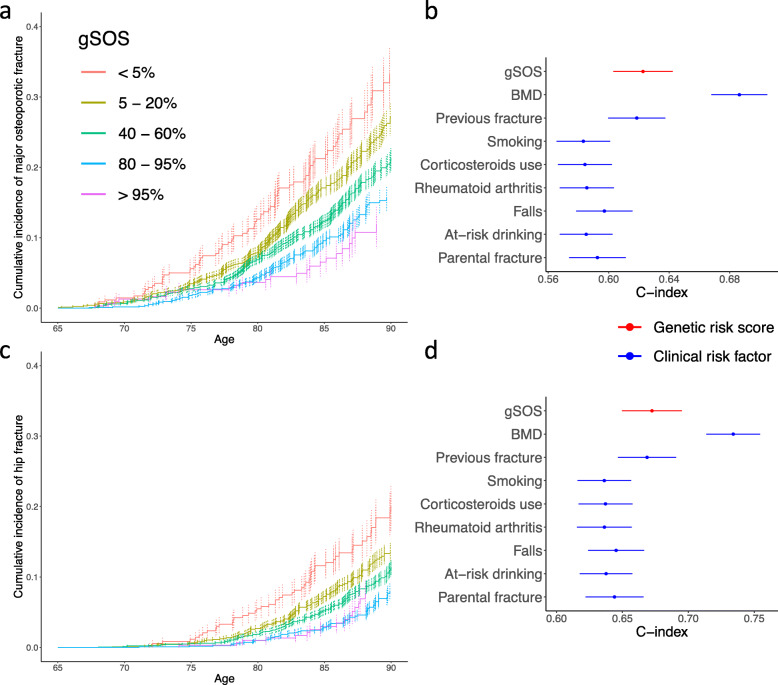
gSOS effectively stratifies cumulative fracture risk. **a** Lower gSOS is associated with a higher cumulative incidence of major osteoporotic fracture. **b** Comparison of the age-dependent predictive performance of gSOS (red) and clinical risk factors (blue) in identifying individuals that experienced incident major osteoporotic fracture. **c** Lower gSOS is associated with a higher cumulative incidence of hip fracture. **d** Comparison of the age-dependent predictive performance of gSOS (red) and clinical risk factors (blue) in identifying individuals that experienced incident hip fracture. All association tests were based on 4663 men from the MrOS US and 3615 women from the SOF cohorts using Cox proportional hazard models adjusted for sex. Only clinical risk factors available in both cohorts are presented. CIs of C-index were derived from 200 bootstrap replicates

Furthermore, gSOS provided a means of stratification of cumulative risk of both incident major osteoporotic fracture and hip fracture. In the MrOS and the SOF cohorts, substantial stratification in cumulative incidence of fracture was observed especially among individuals aged > 75 years (Fig. 3a, c), and this finding was consistent if these cohorts were examined separately (Additional file 1: Fig. S3).

The predictive performance of gSOS also surpassed all clinical risk factors (Fig. 3b, d), except measured femoral neck BMD and prior fracture, with a Cox-model concordance index (C-index) of 0.623 (95% CI 0.603–0.642) for incident major osteoporotic fracture and 0.672 (95% CI 0.650–0.695) for incident hip fracture. We note that the C-index for prior fracture was 0.619 (95% CI 0.600–0.637) for incident major osteoporotic fracture and 0.669 (95% C: 0.647–0.691) for incident hip fracture. Both indices were similar to those of gSOS.

### gSOS improved predictive performance when combined with FRAX clinical risk factors in European populations

We found that combining gSOS with the clinical risk factor-based FRAX (FRAX-CRF) score in all four European ancestry cohorts significantly improved the risk prediction for both incident major osteoporotic fracture and hip fracture (likelihood ratio test *p* values < 0.05; Table 2), without largely modifying the effect size of the FRAX-CRF score. Interestingly, combining gSOS with measured femoral neck BMD or a clinical risk factor and BMD-based FRAX (FRAX-BMD) also significantly enhanced risk prediction (likelihood ratio test *p* values < 0.05; Table 2).

**Table 2:** gSOS is associated with fracture in addition to FRAX-CRF and FRAX-BMD. Odds ratios associated with risk predictors of interest were based on logistic regression

Cohort		UK Biobank test dataset (*N* = 4717)*	MrOS US (*N* = 4663)	MrOS Sweden (*N* = 1880)	SOF (*N* = 3615)
Model	Odds ratio (95% CI)				
**Major osteoporotic fracture**					
Fracture ~ FRAX-CRF	FRAX-CRF^§^	1.15 (1.10–1.20)	1.06 (1.04–1.08)	1.04 (1.02–1.06)	1.04 (1.03–1.05)
Fracture ~ FRAX-CRF + gSOS	FRAX-CRF	1.14 (1.09–1.19)	1.06 (1.04–1.08)	1.04 (1.02–1.06)	1.04 (1.03–1.05)
	gSOS	1.41 (1.18–1.69)	1.36 (1.24–1.49)	1.39 (1.23–1.58)	1.30 (1.19–1.42)
Likelihood ratio test *p* value		1.4 × 10^−4^	3.1 × 10^−11^	6.9 × 10^−8^	2.5 × 10^−9^
Fracture ~ age + sex + BMD	BMD^¶^	1.66 (1.35–2.05)	1.79 (1.64–1.97)	1.86 (1.62–2.15)	2.02 (1.80–2.27)
Fracture ~ age + sex + BMD + gSOS	BMD	1.53 (1.24–1.90)	1.72 (1.56–1.89)	1.79 (1.55–2.07)	1.93 (1.72–2.17)
	gSOS	1.33 (1.11–1.59)	1.22 (1.11–1.34)	1.29 (1.14–1.47)	1.22 (1.11–1.33)
Likelihood ratio test *p* value		2.4 × 10^−3^	3.4 × 10^−5^	6.0 × 10^−5^	2.1 × 10^−5^
Fracture ~ FRAX-BMD	FRAX-BMD^§^	1.17 (1.12–1.22)	1.10 (1.08–1.12)	1.07 (1.05–1.08)	1.06 (1.05–1.07)
Fracture ~ FRAX-BMD + gSOS	FRAX-BMD	1.16 (1.11–1.21)	1.09 (1.08–1.11)	1.06 (1.04–1.08)	1.06 (1.05–1.07)
	gSOS	1.34 (1.12–1.60)	1.28 (1.17–1.40)	1.33 (1.18–1.51)	1.22 (1.12–1.34)
Likelihood ratio test *p* value		1.5 × 10^−3^	1.5 × 10^−7^	4.5 × 10^−6^	1.1 × 10^−5^
**Hip fracture**					
Fracture ~ FRAX-CRF	FRAX-CRF		1.06 (1.04–1.09)	1.02 (0.98–1.05)	1.05 (1.04–1.06)
Fracture ~ FRAX-CRF + gSOS	FRAX-CRF		1.06 (1.04–1.09)	1.02 (0.98–1.05)	1.05 (1.04–1.06)
	gSOS		1.36 (1.20–1.54)	1.25 (1.04–1.50)	1.31 (1.19–1.44)
Likelihood ratio test *p* value			1.1 × 10^−6^	1.6 × 10^−2^	4.2 × 10^−8^
Fracture ~ age + sex + BMD	BMD		2.08 (1.83–2.38)	2.45 (1.96–3.08)	2.04 (1.80–2.32)
Fracture ~ age + sex + BMD + gSOS	BMD		2.01 (1.76–2.31)	2.41 (1.92–3.04)	1.95 (1.72–2.22)
	gSOS		1.17 (1.03–1.33)	1.11 (0.92–1.34)	1.20 (1.08–1.32)
Likelihood ratio test *p* value			1.7 × 10^−2^	2.7 × 10^−1^	3.7 × 10^−4^
Fracture ~ FRAX-BMD	FRAX-BMD		1.10 (1.08–1.13)	1.06 (1.04–1.09)	1.07 (1.05–1.08)
Fracture ~ FRAX-BMD + gSOS	FRAX-BMD		1.10 (1.07–1.12)	1.06 (1.04–1.09)	1.06 (1.05–1.08)
	gSOS		1.31 (1.15–1.48)	1.20 (1.00–1.44)	1.25 (1.13–1.37)
Likelihood ratio test *p* value			3.0 × 10^−5^	5.3 × 10^−2^	1.1 × 10^−5^

*Only 4717 individuals in the UK Biobank had measured femoral neck BMD and were included; 125 individuals experienced incident major osteoporotic fracture; only one individual experienced incident hip fracture; thus, model comparison was not conducted

^§^Based on one unit (percent of 10-year fracture probability) increase in FRAX-CRF or FRAX-BMD

^¶^Based on one standard deviation decrease in measured femoral neck BMD

**Fig. 4:**
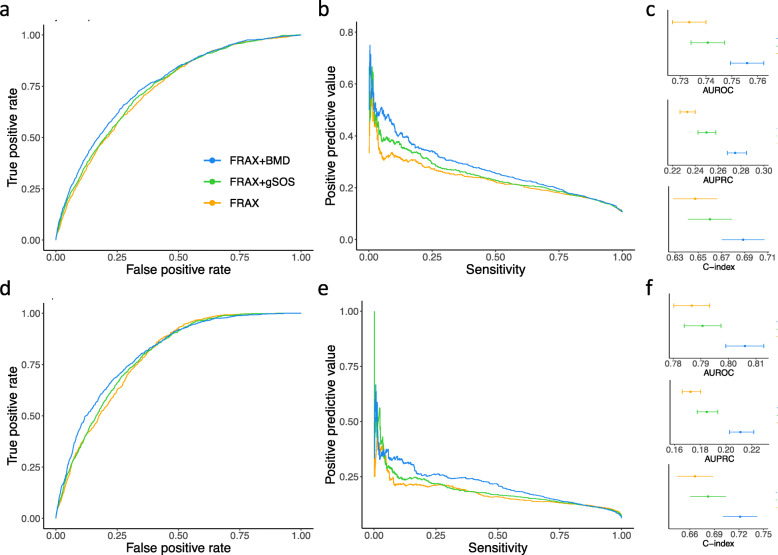
Comparison of the predictive performance of FRAX-CRF, FRAX-BMD, and FRAX-gSOS. **a** Receiver operating characteristic curves, **b** precision-recall curves, and **c** summaries of the predictive performance of FRAX-based scores for predicting incident major osteoporotic fracture risk. **d** Receiver operating characteristic curves, **e** precision-recall curves, and **f** summaries of the predictive performance of FRAX-based scores for predicting incident hip fracture risk. AUROC and AUPRC were derived based on all 90,172 individuals from four cohorts. C-indices were derived based on 4663 men from the MrOS US and 3615 women from the SOF cohorts using Cox proportional hazard models. All CIs were derived from 200 bootstrap replicates

We therefore developed a combined score including gSOS and the FRAX score with clinical risk factors (FRAX-gSOS; the “Methods” section). Although the FRAX-BMD score remained the best predictor for both major osteoporotic fracture (AUROC 0.756 (95% CI 0.749–0.762); AUPRC 0.275 (95% CI 0.268–0.285); C-index 0.689 (95% CI 0.670–0.707)) and hip fracture (AUROC 0.806 (95% CI 0.799–0.813); AUPRC 0.211 (95% CI 0.203–0.222); C-index 0.722 (95% CI 0.701–0.742)), the FRAX-gSOS score had a modestly improved predictive performance compared to the FRAX-CRF score. Specifically, the FRAX-gSOS offered a higher true positive rate at most given false positive rates, as well as greater positive predictive value at most levels of sensitivity, as indicated by the ROCs and PRCs (Fig. 4a, b, d, e) although the magnitude of these differences was not large. Correspondingly, the FRAX-gSOS score had a modestly higher AUROC (0.741 (95% CI 0.734–0.737) vs. 0.733 (95% CI 0.727–0.740), DeLong’s test *p* value = 1.9 × 10^−2^, for major osteoporotic fracture; 0.791 (95% CI 0.784–0.797) vs. 0.787 (95% CI 0.780–0.793), DeLong’s test *p* value = 0.23, for hip fracture), AUPRC (0.250 (95% CI 0.242–0.258) vs. 0.233 (95% CI 0.227–0.240) for major osteoporotic fracture; 0.185 (95% CI 0.178–0.194) vs. 0.172 (95% CI 0.166–0.180) for hip fracture), and C-index (0.660 (95% CI 0.641–0.679) vs. 0.647 (95% CI 0.603–0.642) for major osteoporotic fracture; 0.682 (95% CI 0.660–0.704) vs. 0.666 (95% CI 0.644–0.688) for hip fracture) than the FRAX-CRF score (Fig. 4c, f).

In all four study cohorts, including gSOS in the FRAX score generally increased the predicted probability of future fracture (Table 1), and more individuals were classified as having higher major osteoporotic fracture risk or hip fracture risk (Table 3, Additional file 1: Table S5, and Additional file 1: Table S6). Furthermore, the positive overall NRIs and IDIs suggested that this could refine the risk screening (Table 3, Additional file 1: Table S5, and Additional file 1: Table S6). For instance, adding gSOS to the FRAX score refined the prediction of major osteoporotic fracture risk with NRIs being 0.028 in the UK Biobank test dataset, 0.043 in the MrOS US, 0.072 in the MrOS Sweden, and 0.024 in the SOF. Improvements were also observed for re-classifying the risk of hip fracture across all cohorts, with the exception that, in the SOF cohort, FRAX-gSOS did not better identify individuals at higher risk of hip fracture.

**Table 3:** Net reclassification improvement of fracture risk prediction using FRAX-gSOS

Cohort	UKB*	US^§^	SWE^¶^	SOF^†^	UKB	US	SWE	SOF
	Major osteoporotic fracture				Hip fracture			
\Categorical NRI (95% CI)	0.028 (0.016–0.039)	0.043 (0.020–0.066)	0.072 (0.027–0.118)	0.024 (− 0.008–0.056)	0.055 (0.009–0.100)	0.066 (0.025–0.107)	0.037 (− 0.012–0.085)	− 0.001 (− 0.034–0.031)
Continuous NRI (95% CI)	0.300 (0.245–0.354)	0.179 (0.092–0.267)	0.232 (0.116–0.348)	0.237 (0.155–0.318)	0.206 (0.073–0.339)	0.180 (0.060–0.300)	0.132 (− 0.045–0.310)	0.205 (0.116–0.295)
IDI (95% CI)	0.010 (0.008–0.011)	0.011 (0.008–0.015)	0.014 (0.009–0.019)	0.017 (0.012–0.021)	0.003 (0.001–0.005)	0.007 (0.003–0.011)	0.004 (− 0.002–0.011)	0.011 (0.007–0.015)

*The UK Biobank test dataset (*N* = 80,014)

^§^The MrOS US cohort (*N* = 4663)

^¶^The MrOS Sweden cohort (*N* = 1880)

^†^The SOF cohort (*N* = 3615)

### gSOS was associated with fracture risk at an attenuated magnitude in an Asian population

A standard deviation decrease in gSOS was associated with an increased odds of incident major osteoporotic fracture in the UK Biobank, MrOS US, MrOS Sweden, and SOF cohorts, ranging from 1.35 to 1.46 (Table 4). In contrast, this decrease was associated with an attenuated 1.26-fold (95% CI 1.13–1.41) increased odds of incident major osteoporotic fracture in the CKB cohort (Table 4). Similarly, the association between a decrease in gSOS and increase in incident hip fracture risk was also slightly smaller in the CKB cohort, compared to those of European ancestry (Table 4). This attenuation was not modified by consideration of other risk factors (Table 4).

**Table 4:** Odds ratios (OR) for the association between per standard deviation decrease in gSOS and increased odds of incident fracture

	OR (95% CI)*	*p* value	OR (95% CI), adjusted for RFs^§^	*p* value, adjusted for RFs
UK Biobank test dataset (*N* = 80,014)				
Major osteoporotic fracture	1.46 (1.38–1.55)	1.1 × 10^−37^	1.42 (1.34–1.51)	1.4 × 10^−32^
Hip fracture	1.37 (1.19–1.57)	6.3 × 10^−6^	1.34 (1.16–1.53)	3.7 × 10^−5^
MrOS US (*N* = 4663)				
Major osteoporotic fracture	1.38 (1.26–1.51)	2.2 × 10^−12^	1.31 (1.17–1.45)	1.1 × 10^−6^
Hip fracture	1.37 (1.21–1.56)	5.5 × 10^−7^	1.33 (1.15–1.55)	1.4 × 10^−4^
MrOS Sweden (*N* = 1880)				
Major osteoporotic fracture	1.45 (1.28–1.65)	5.3 × 10^−9^	1.38 (1.17–1.63)	1.3 × 10^−4^
Hip fracture	1.31 (1.08–1.58)	5.0 × 10^−3^	1.35 (1.05–1.72)	1.8 × 10^−2^
SOF (*N* = 3615)				
Major osteoporotic fracture	1.35 (1.24–1.47)	1.0 × 10^−11^	1.34 (1.21–1.48)	7.3 × 10^−9^
Hip fracture	1.31 (1.19–1.43)	1.4 × 10^−8^	1.32 (1.19–1.47)	4.1 × 10^−7^
CKB (*N* = 25,034)				
Major osteoporotic fracture	1.26 (1.13–1.41)	5.3 × 10^−5^	1.22 (1.09–1.36)	6.8 × 10^−4^
Hip fracture	1.25 (0.98–1.60)	7.1 × 10^−2^	1.21 (0.94–1.54)	0.14

*Derived from logistic regression models adjusted for age and sex

^§^Derived from logistic regression models adjusted for age, sex and other available risk factors (RFs) listed in Table 1

## Discussion

Here, we generated a genome-wide polygenic risk score for SOS, gSOS, from 341,449 individuals of European ancestry and tested its performance characteristics for predicting incident fracture in four European ancestry cohorts comprising 90,172 individuals. While the source population, the UK Biobank, which we used to develop the polygenic risk score was not completely representative of a general population, we provide evidence that gSOS, particularly at its extremes, is consistently associated with risk of major osteoporotic and hip fracture across multiple cohorts. Since gSOS does not capture all variance in measured BMD, it does not predict fracture as accurately as the latter. However, gSOS has a moderately higher associated odds of incident fracture and a better predictive performance of incident fracture risk than many traditional clinical risk factors. Indeed, gSOS remained associated with incident fracture, even after accounting for FRAX-CRF and FRAX-BMD. Further, gSOS improved the clinical risk factor-based FRAX score and resulted in modestly better risk stratification for fracture.

By summarizing genetic predisposition conferred by polymorphisms throughout the human genome, polygenic risk scores can now be tested for their ability to predict common disease outcomes and their risk factors. For instance, a polygenic risk score for breast cancer is able to identify 10% of the population having a 32.6% lifetime risk [47]. These polygenic risk scores can be generated from one single investment in genome-wide genotyping, the cost of which is now approximately $40 USD in a research context [48]. However, sample collection, DNA extraction, and technical support will also incur additional costs. Though technologies for measuring BMD, such as DXA scan, are well-developed and of clinical utility [49], their cost and restricted accessibility currently limits risk screening to only a proportion of the population. Therefore, as polygenic risk scores continue to improve, with larger sample sizes providing better estimates of SOS and BMD, such polygenic risk scores may find a clinical utility to assist in fracture prediction.

Using FRAX-gSOS modestly improved predictive performance over a FRAX-CRF score. Specifically, a small proportion of the population at high fracture risk were correctly re-classified using FRAX-gSOS. While FRAX-gSOS does not perform as well as FRAX-BMD, given the low incremental cost of generating gSOS in individuals already genome-wide genotyped, FRAX-gSOS may improve risk prediction in the proportion of the population for which BMD is not measured. As sample sizes increase for genomic studies, we expect that this predictive ability will further improve. Besides, already gSOS provides information above FRAX-BMD (Table 2), in terms of fracture prediction.

Our study highlights several directions for future studies. First, further improved polygenic risk scores for BMD, in addition to SOS, may be pursued, since BMD is a well-used metric of fracture risk that is incorporated into the FRAX algorithm [6, 50]. It should be noted that genetic risk scores for BMD based on polymorphisms affecting several pivotal BMD genes have already been developed and shown to be able to predict fracture risk from different perspectives [16, 17, 21]. However, in order to generate more accurate genome-wide polygenic risk scores that could possibly capture a larger proportion of total variance in BMD, very large sample sizes of genome-wide genotyped individuals are required. Such resources do not currently exist for BMD. We anticipate this will improve as more individuals are genome-wide genotyped with BMD measures. Second, gSOS was developed among white British individuals and the cohorts employed in this study include predominantly individuals of European ancestry. As has been widely noted, when a polygenic risk score is applied to populations of different ancestries, its predictive performance may vary, sometimes to a large extent [51]. For instance, genetic risk factors for osteoporotic fracture and BMD characterized in European populations do not improve the prediction of fracture risk in a Chinese population [52]. Here, although gSOS was still significantly associated with both incident major osteoporotic fracture risk and hip fracture risk in the CKB cohort, the magnitude of the association was attenuated. Therefore, we think that gSOS should not be directly applied to populations with a non-European ancestry, especially since a large number of SNPs in gSOS were not present in the targeted population, due to different imputation reference panels and, potentially, changes in minor allele frequencies. However, we expect that upcoming studies developing population-specific polygenic risk scores, which might properly model linkage disequilibrium, minor allele frequencies, population-specific effect sizes, and heritability [53], will be able to address this important issue. Lastly, it should be noted that gSOS yielded predictive and discriminative power while being independent of most fracture risk factors. While we endeavored to combine gSOS and other risk factors through FRAX-gSOS for improved prediction, more rigorous approaches may warrant further research. Meanwhile, as has been done for the FRAX clinical risk score, absolute risks of fracture associated with FRAX-gSOS should be calibrated in a population-specific manner, accounting for national differences in fracture rates.

## Conclusions

In conclusion, we generated and validated a polygenic risk score for SOS which is consistently associated with the risk of fracture. While this score provides modestly better fracture risk discrimination than many clinical risk factors, it does not perform as well as BMD. However, gSOS should be explored as a tool to provide better risk stratification to identify individuals at high risk of fracture, particularly considering that polygenic risk scores can be generated for multiple diseases from one single investment in genome-wide genotyping.

## Supplementary Information

**Additional file 1:** **Fig. S1**: Distribution of discrepancies in minor allele frequencies of SNPs between the China Kadoorie Biobank and the UK Biobank; **Fig. S2**: Distribution of standardized gSOS among 90,172 individuals of European ancestry; **Fig. S3**: Cumulative incidence of major osteoporotic fracture in (A) the MrOS US cohort and (B) the SOF cohort, and cumulative incidence of hip fracture in (C) the MrOS US cohort and (D) the SOF cohort; **Table S1**. Summary of model training and selection in the UK Biobank; **Table S2**. Summary of Pearson correlation between gSOS and clinical risk factors; **Table S3**. Summary of incidence of osteoporotic fracture; **Table S4**. Summary of predictive power of gSOS and clinical risk factors; **Table S5**. Details of net reclassification improvement of major osteoporotic fracture risk prediction using FRAX-gSOS; **Table S6**. Details of net reclassification improvement of hip fracture risk prediction using FRAX-gSOS.

## Data Availability

Data that support the findings of this study are available from the UK Biobank [25], the MrOS US cohort [26], the MrOS Sweden cohort [27], the SOF cohort [28, 29], and the China Kadoorie Biobank [30], but restrictions apply to the availability of these data, which were used under license for the current study, and so are not publicly available. Data are however available upon successful project applications to respective research committees. gSOS has been deposited on the PGS catalog (https://www.pgscatalog.org/score/PGS000657/).

## Abbreviations

AUPRC: Area under the precision-recall curve
AUROC: Area under the receiver operating characteristic curve
BMD: Bone mineral density
CI: Confidence interval
CKB: China Kadoorie Biobank
CRF: Clinical risk factor
DXA: Dual-energy X-ray absorptiometry
FRAX: Fracture Risk Assessment Tool
gSOS: Genomic speed of sound
HRC: Haplotype Reference Consortium
IDI: Integrated discrimination index
LASSO: Least absolute shrinkage and selection operator
MrOS Sweden: Sweden-based Osteoporotic Fractures in Men Study
MrOS US: United States-based Osteoporotic Fractures in Men Study
NRI: Net reclassification index
OR: Odds ratio
SNP: Single nucleotide polymorphism
SOF: Study of Osteoporotic Fractures
SOS: Speed of sound
