# Supplementary material for: Improved prediction of fracture risk leveraging a genome-wide polygenic risk score
Source: Genome Med. 2021 Feb 3;13:16. doi: 10.1186/s13073-021-00838-6 (PMC7860212; doi:10.1186/s13073-021-00838-6)
Supplement: Supplementary file 1 — Additional file 1: Fig. S1: Distribution of discrepancies in minor allele frequencies of SNPs between the China Kadoorie Biobank and the UK Biobank; Fig. S2: Distribution of standardized gSOS among 90,172 individuals of European ancestry; Fig. S3: Cumulative incidence of major osteoporotic fracture in (A) the MrOS US cohort and (B) the SOF cohort, and cumulative incidence of hip fracture in (C) the MrOS US cohort and (D) the SOF cohort; Table S1. Summary of model training and selection in the UK Biobank; Table S2. Summary of Pearson correlation between gSOS and clinical risk factors; Table S3. Summary of incidence of osteoporotic fracture; Table S4. Summary of predictive power of gSOS and clinical risk factors; Table S5. Details of net reclassification improvement of major osteoporotic fracture risk prediction using FRAX-gSOS; Table S6. Details of net reclassification improvement of hip fracture risk prediction using FRAX-gSOS. [file 13073_2021_838_MOESM1_ESM.pdf]

Supplementary Materials

Improved prediction of fracture risk leveraging a genome-wide polygenic risk score

Lu, T. et al.

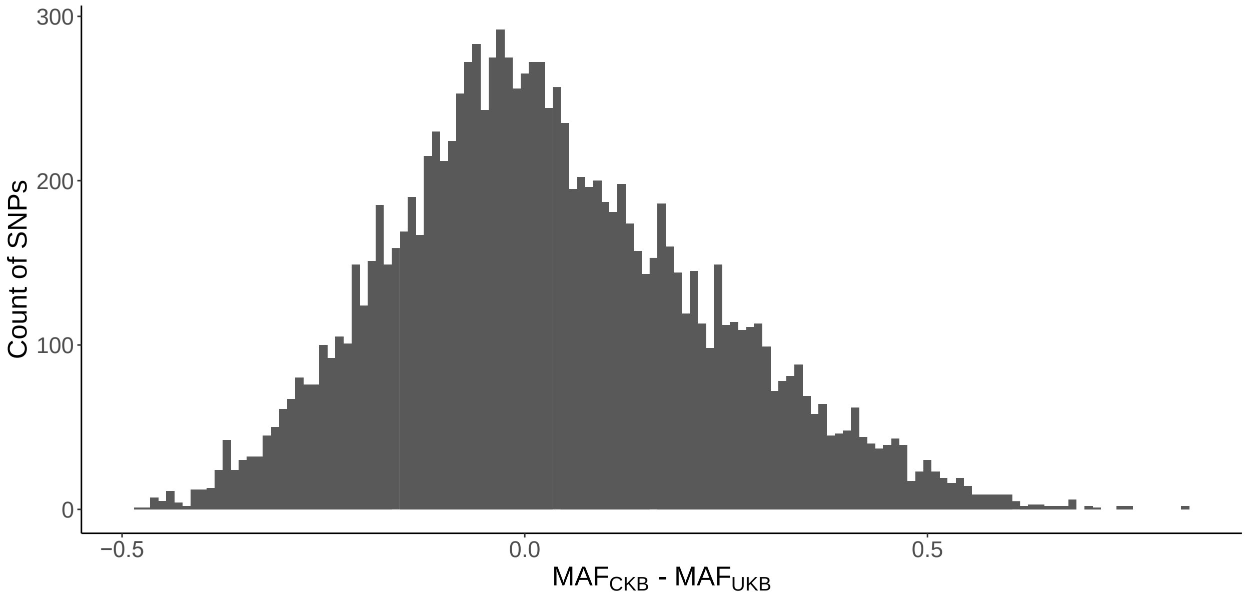

Fig. S1. Distribution of discrepancies in minor allele frequencies of SNPs between the China Kadoorie Biobank (CKB) and the UK Biobank. The distribution was derived based on 13,848 gSOS SNPs available in CKB. Minor allele frequency discrepancy may exceed 0.5 because some SNPs had reversed minor and major alleles in these two cohorts.

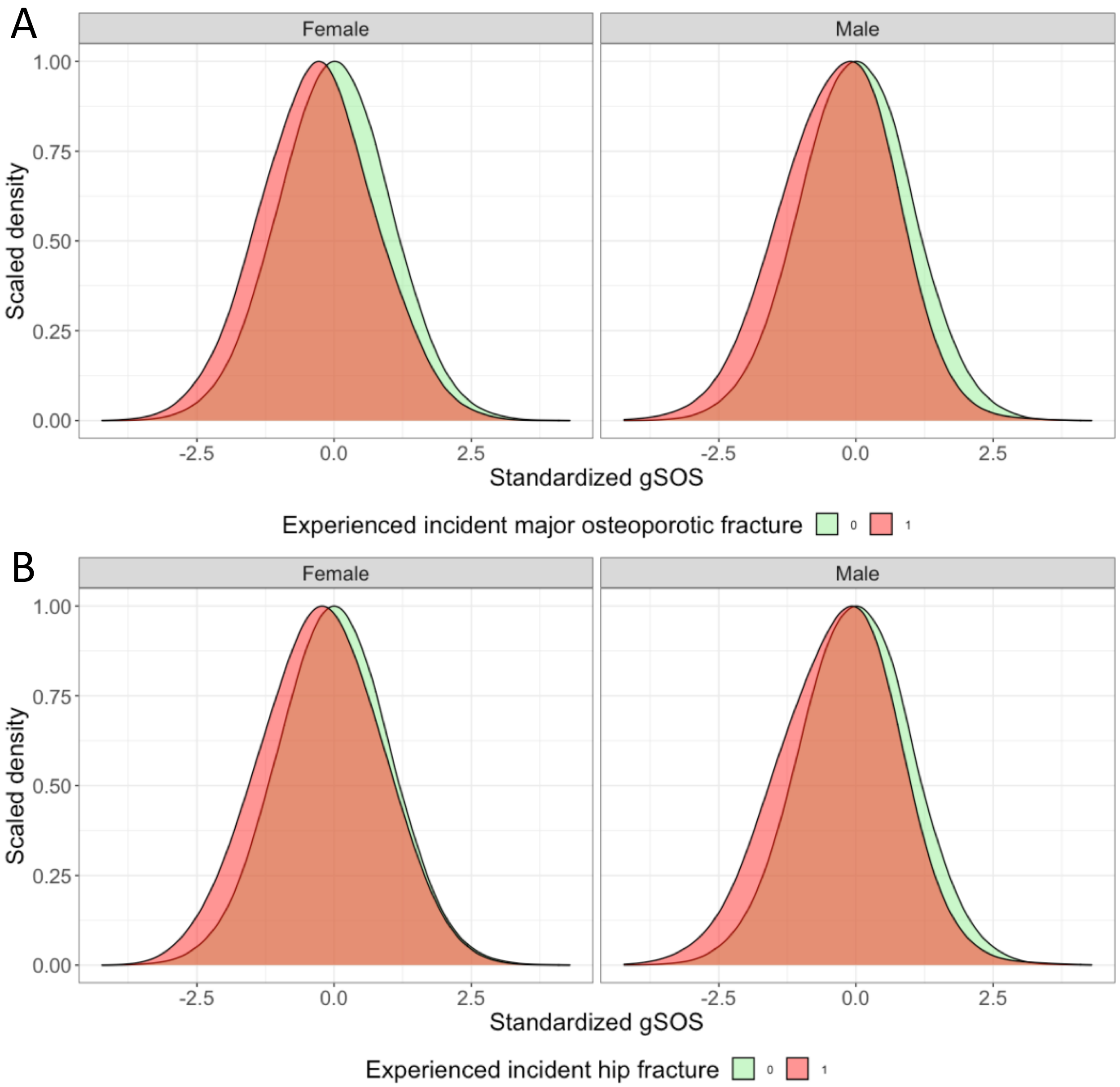

Fig. S2. Distribution of standardized gSOS among 90,172 individuals of European ancestry. Individuals who experienced incident major osteoporotic fracture or incident hip fracture had significantly lower standardized gSOS than those who did not experience incident fracture. This trend was consistent in both women (t-test p-value = 4.5×10^{-33} for major osteoporotic fracture; 1.0×10^{-9} for hip fracture) and men (t-test p-value = 1.8×10^{-23} for major osteoporotic fracture; 1.7×10^{-9} for hip fracture).

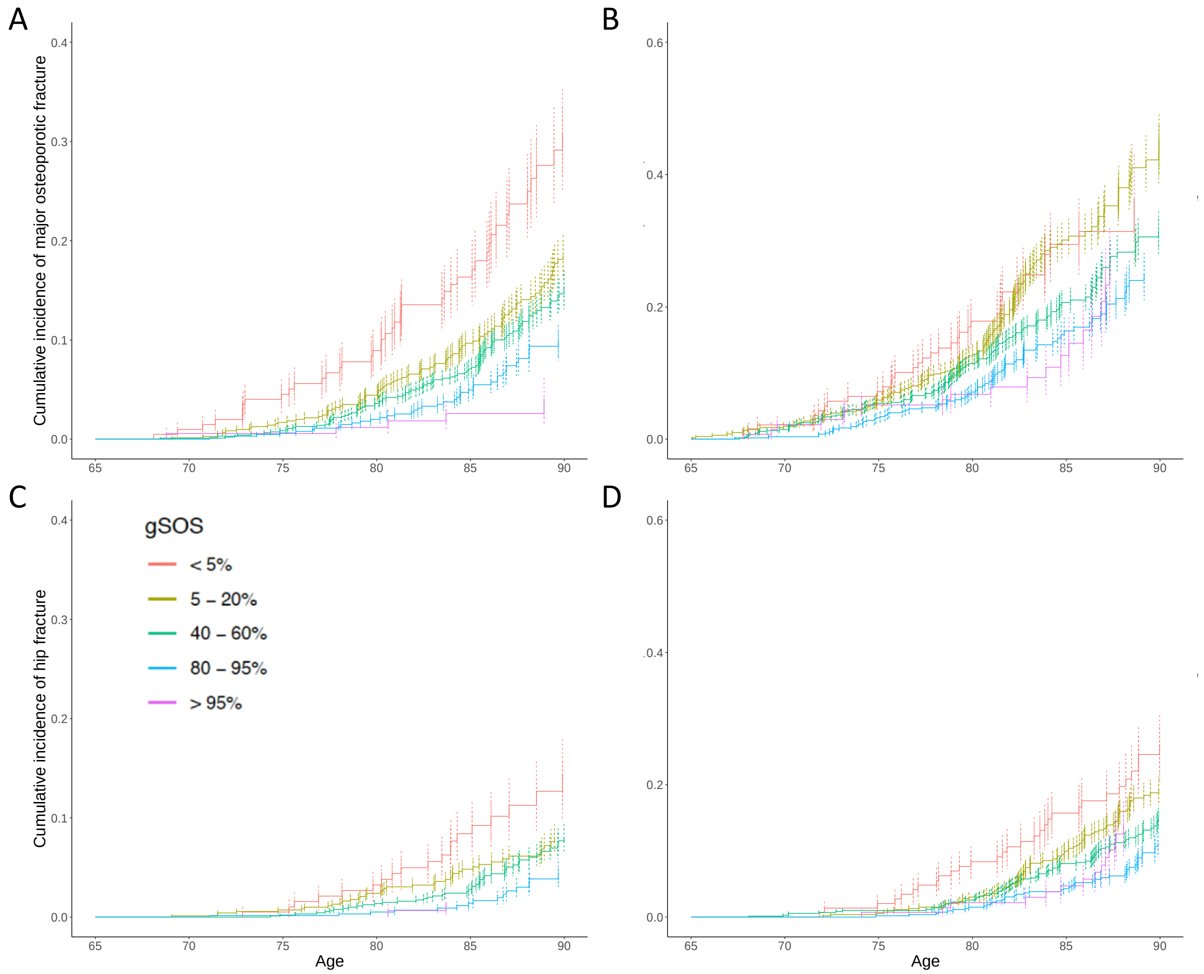

Fig. S3. Cumulative incidence of major osteoporotic fracture in (A) the MrOS US cohort and (B) the SOF cohort, and cumulative incidence of hip fracture in (C) the MrOS US cohort and (D) the SOF cohort. Both cohorts enrolled elderly men or women aged ≥ 65 years. Kaplan-Meier estimates were used to estimate the cumulative incidence, censored at 90 years.

Table S1: Summary of model training and selection in the UK Biobank. This table is adapted from supplementary Table S3 in Forgetta et al [1].

GWAS* p-value threshold	$5x10^{-3}$	$5x10^{-4}$	$5x10^{-5}$	$5x10^{-6}$	$5x10^{-7}$	$5x10^{-8}$
No. of total SNPs	642,127	345,111	227,478	166,576	130,130	104,836
No. of activated SNPs by LASSO	40,864	21,717	13,793	10,348	7,999	6,823
λ parameter§	0.0032	0.0028	0.0021	0.0016	0.0015	0.0013
Proportion of variance explained¶	24.53%	24.99%	24.89%	24.75%	23.86%	23.38%
Lower bound of 95% CI	22.54%	22.99%	22.89%	22.75%	21.88%	21.41%
Upper bound of 95% CI	26.55%	27.02%	26.91%	26.77%	25.87%	25.38%

* Genome-wide association study performed in the training dataset (N = 341,449)

§ λ in the LASSO model optimized in the model selection dataset (N = 5,335)

¶ Highest proportion of variance explained achieved in the model selection dataset with optimized λ

Table S2: Summary of Pearson correlation between gSOS and clinical risk factors.

Cohort	UK Biobank Test Dataset (N = 80,014)	MrOS US (N = 4,663)	MrOS Sweden (N = 1,880)		SOF (N = 3,615)	CKB (N = 25,034)
			Malmö	Gothenburg		
Age	0.01 (0.00 – 0.01)*	0.00 (-0.03 – 0.03)	0.02 (-0.05 – 0.08)	-0.04 (-0.10 – 0.03)	0.02 (-0.01 – 0.06)	0.01 (0.00 – 0.02)
Sex	0.00 (-0.01 – 0.00)	NA	NA	NA	NA	0.01 (0.00 – 0.02)
Baseline BMI	0.00 (0.00 – 0.01)	0.02 (-0.01 – 0.05)	0.01 (-0.05 – 0.08)	0.05 (-0.01 – 0.11)	0.00 (-0.04 – 0.03)	-0.01 (-0.02 – 0.00)
Prior fracture	-0.07 (-0.08 – -0.06)	-0.09 (-0.12 – -0.06)	-0.09 (-0.16 – -0.03)	-0.09 (-0.15 – -0.02)	-0.12 (-0.15 – -0.09)	-0.05 (-0.06 – -0.04)
Smoking	0.00 (-0.01 – 0.01)	-0.04 (-0.07 – -0.01)	-0.08 (-0.14 – -0.01)	0.02 (-0.04 – 0.08)	0.01 (-0.02 – 0.04)	0.00 (-0.01 – 0.01)
Corticosteroids use	0.00 (-0.01 – 0.01)	0.01 (-0.02 – 0.04)	0.00 (-0.07 – 0.06)	-0.03 (-0.09 – 0.03)	0.05 (0.02 – 0.09)	NA
Rheumatoid arthritis	-0.01 (-0.01 – 0.00)	0.01 (-0.01 – 0.04)	-0.03 (-0.09 – 0.04)	0.04 (-0.02 – 0.10)	0.00 (-0.03 – 0.03)	0.01 (0.00 – 0.02)
Parental fracture	NA	-0.05 (-0.09 – -0.02)	-0.10 (-0.18 – -0.01)	0.02 (-0.06 – 0.10)	-0.05 (-0.09 – -0.01)	NA
At-risk drinking	NA	-0.01 (-0.04 – 0.02)	0.01 (-0.05 – 0.08)	-0.02 (-0.08 – 0.05)	-0.01 (-0.04 – 0.02)	NA
Falls	NA	-0.01 (-0.04 – 0.02)	0.01 (-0.06 – 0.07)	-0.04 (-0.10 – 0.02)	0.00 (-0.04 – 0.03)	NA
Secondary osteoporosis	0.00 (-0.01 – 0.00)	NA	NA	NA	NA	NA

* Pearson correlation (95% CI)

Table S3: Summary of incidence of osteoporotic fracture.

Cohort	UK Biobank Test Dataset (N = 80,014)	MrOS US (N = 4,663)	MrOS Sweden (N = 1,880)	SOF (N = 3,615)
**Major osteoporotic fracture (%)**				
Total	1,189 (1.5)	560 (12.0)	337 (17.9)	707 (20.6)
gSOS range				
< 1%	25 (3.1)	11 (26.8)	9 (47.4)	9 (33.3)
1-5%	79 (2.5)	44 (21.6)	20 (26.7)	37 (26.6)
5-20%	269 (2.2)	104 (14.4)	61 (21.6)	134 (26.3)
20-40%	266 (1.7)	123 (13.0)	71 (18.8)	168 (24.6
40-60%	251 (1.6)	111 (11.6)	81 (21.5)	136 (19.9)
60-80%	176 (1.1)	109 (11.7)	49 (13.0)	116 (16.9)
80-95%	103 (0.9)	48 (7.4)	40 (14.2)	85 (16.2)
95-99%	17 (0.5)	8 (4.6)	6 (8.0)	20 (14.8)
≥ 99%	3 (0.4)	2 (5.6)	0 (0)	2 (5.4)
**Hip fracture (%)**				
Total	209 (0.3)	273 (5.9)	129 (6.9)	556 (15.6)
gSOS range				
< 1%	7 (0.9)	7 (17.1)	3 (15.8)	6 (20.0)
1-5%	10 (0.3)	20 (9.8)	5 (6.7)	36 (24.7)
5-20%	47 (0.4)	51 (7.1)	23 (8.2)	100 (18.7)
20-40%	40 (0.3)	55 (5.8)	26 (6.9)	128 (17.9)
40-60%	42 (0.3)	59 (6.2)	37 (9.8)	102 (14.4)
60-80%	36 (0.2)	56 (6.0)	18 (4.8)	102 (14.4)
80-95%	25 (0.2)	20 (3.1)	15 (5.3)	63 (11.7)
95-99%	2 (0.1)	4 (2.3)	2 (2.7)	17 (12.2)
≥ 99%	0 (0)	1 (2.8)	0 (0)	2 (5.1)

Table S4: Summary of predictive power (Area Under the Receiver Operating Characteristic curve) of gSOS and clinical risk factors.

Cohort[*]	UK Biobank Test Dataset (N = 80,014)[§]	MrOS US (N = 4,663)[¶]	MrOS Sweden (N = 1,880)[¶]	SOF (N = 3,615)[¶]
**Major osteoporotic fracture (95% CI)**				
gSOS	0.680 (0.673 – 0.688)	0.621 (0.609 – 0.633)	0.597 (0.576 – 0.618)	0.629 (0.617 – 0.640)
Prior fracture	0.667 (0.660 – 0.675)	0.616 (0.603 – 0.628)	0.560 (0.538 – 0.582)	0.629 (0.618 – 0.641)
Smoking	0.654 (0.646 – 0.661)	0.591 (0.579 – 0.603)	0.556 (0.535 – 0.577)	0.607 (0.596 – 0.619)
Corticosteroids use	0.651 (0.643 – 0.659)	0.587 (0.574 – 0.599)	0.548 (0.526 – 0.570)	0.604 (0.593 – 0.616)
Rheumatoid arthritis	0.653 (0.646 – 0.661)	0.594 (0.582 – 0.606)	0.548 (0.527 – 0.570)	0.606 (0.594 – 0.617)
Parental fracture	NA	0.597 (0.585 – 0.610)	0.550 (0.528 – 0.572)	0.607 (0.595 – 0.618)
At-risk drinking	NA	0.603 (0.590 – 0.617)	0.549 (0.527 – 0.570)	0.600 (0.588 – 0.613)
Falls	NA	0.592 (0.580 – 0.604)	0.584 (0.562 – 0.606)	0.604 (0.592 – 0.615)
Secondary osteoporosis	0.653 (0.645 – 0.661)	NA	NA	NA
**Hip fracture (95% CI)**				
gSOS	0.739 (0.724 – 0.754)	0.651 (0.635 – 0.667)	0.588 (0.559 – 0.616)	0.617 (0.605 – 0.630)
Prior fracture	0.732 (0.716 – 0.748)	0.639 (0.623 – 0.655)	0.564 (0.534 – 0.594)	0.615 (0.603 – 0.627)
Smoking	0.728 (0.712 – 0.745)	0.637 (0.621 – 0.653)	0.567 (0.536 – 0.599)	0.597 (0.585 – 0.610)
Corticosteroids use	0.728 (0.711 – 0.744)	0.632 (0.606 – 0.640)	0.572 (0.542 – 0.601)	0.595 (0.583 – 0.608)
Rheumatoid arthritis	0.732 (0.715 – 0.749)	0.629 (0.612 – 0.645)	0.565 (0.535 – 0.595)	0.597 (0.585 – 0.610)
Parental fracture	NA	0.631 (0.615 – 0.648)	0.572 (0.540 – 0.603)	0.597 (0.585 – 0.610)
At-risk drinking	NA	0.640 (0.621 – 0.659)	0.565 (0.535 – 0.595)	0.598 (0.584 – 0.612)
Falls	NA	0.628 (0.612 – 0.644)	0.580 (0.548 – 0.611)	0.596 (0.584 – 0.609)
Secondary osteoporosis	0.726 (0.710 – 0.742)	NA	NA	NA

[*] Based on all individuals with available information

[§] Derived from logistic regression models adjusted for age and sex

[¶] Derived from logistic regression models adjusted for age

Table S5: Details of net reclassification improvement of major osteoporotic fracture risk prediction using FRAX-gSOS.

Cohort	UKB*	US§	SWE¶	SOF†	UKB	US	SWE	SOF	UKB	US	SWE	SOF
**Outcome: no fracture**												
	FRAX-gSOS											
FRAX	[0,20)				[20,100]				% reclassified			
[0,20)	77,932	3,823	1,299	1,663	647	144	130	206	1	4	9	11
[20,100]	58	28	22	153	188	102	92	698	24	22	19	18
**Outcome: fracture**												
	FRAX-gSOS											
FRAX	[0,20)				[20,100]				% reclassified			
[0,20)	1,119	491	243	296	46	41	55	70	4	8	18	19
[20,100]	4	1	7	39	20	27	32	302	17	4	18	11
**Combined**												
	FRAX-gSOS											
FRAX	[0,20)				[20,100]				% reclassified			
[0,20)	79,051	4,314	1542	2,022	693	185	185	298	1	4	11	13
[20,100]	62	29	29	204	208	129	124	1,053	23	18	19	16

* The UK Biobank Test Dataset (N = 80,014)

§ The MrOS US cohort (N = 4,663)

¶ The MrOS Sweden cohort (N = 1,880)

† The SOF cohort (N = 3,615)

Table S6: Details of net reclassification improvement of hip fracture risk prediction using FRAX-gSOS.

Cohort	UKB*	US§	SWE¶	SOF†	UKB	US	SWE	SOF	UKB	US	SWE	SOF
**Outcome: no fracture**												
	FRAX-gSOS											
FRAX	[0,3)				[3,100]				% reclassified			
[0,3)	75,729	2,170	125	976	1,885	319	57	201	2	13	31	17
[3,100]	510	319	162	240	1,681	1,576	1,407	1587	23	17	10	13
**Outcome: fracture**												
	FRAX-gSOS											
FRAX	[0,3)				[3,100]				% reclassified			
[0,3)	154	79	5	87	20	25	3	31	11	24	38	26
[3,100]	5	7	6	39	30	162	115	399	14	4	5	9
**Combined**												
	FRAX-gSOS											
FRAX	[0,3)				[3,100]				% reclassified			
[0,3)	75,883	2,249	130	1,069	1,905	344	60	233	2	13	32	18
[3,100]	515	326	168	280	1,711	1,738	1,522	1,995	23	16	10	12

* The UK Biobank Test Dataset (N = 80,014)
§ The MrOS US cohort (N = 4,663)
¶ The MrOS Sweden cohort (N = 1,880)
† The SOF cohort (N = 3,615)

Additional References

1. Forgetta, V., et al., *Machine Learning to Predict Osteoporotic Fracture Risk from Genotypes.* bioRxiv, 2018: p. 413716.
